# Depth Structure from Asymmetric Shading Supports Face Discrimination

**DOI:** 10.1371/journal.pone.0055865

**Published:** 2013-02-14

**Authors:** Chien-Chung Chen, Chin-Mei Chen, Christopher W. Tyler

**Affiliations:** 1 Department of Psychology, National Taiwan University, Taipei, Taiwan; 2 Neurobiology and Cognitive Science Center, National Taiwan University, Taipei, Taiwan; 3 The Smith-Kettlewell Eye Research Institute, San Francisco, California, United States of America; University of Montreal, Canada

## Abstract

To examine the effect of illumination direction on the ability of observers to discriminate between faces, we manipulated the direction of illumination on scanned 3D face models. In order to dissociate the surface reflectance and illumination components of front-view face images, we introduce a symmetry algorithm that can separate the symmetric and asymmetric components of the face in both low and high spatial frequency bands. Based on this approach, hybrid faces stimuli were constructed with different combinations of symmetric and asymmetric spatial content. Discrimination results with these images showed that asymmetric illumination information biased face perception toward the structure of the shading component, while the symmetric illumination information had little, if any, effect. Measures of perceived depth showed that this property increased systematically with the asymmetric but not the symmetric low spatial frequency component. Together, these results suggest that (1) the asymmetric 3D shading information dramatically affects both the perceived facial information and the perceived depth of the facial structure; and (2) these effects both increase as the illumination direction is shifted to the side. Thus, our results support the hypothesis that face processing has a strong 3D component.

## Introduction

The visual system allows us to perceive a 3D representation of an object from the 2D retinal image, even when viewing with one eye (or looking at a 2D photograph). One of the useful cues to this 3D representation is shading, or the gradual variation of the luminance of an illuminated surface [1]. The luminance reaching the eye from a point on a surface depends jointly on the intensity and the incident angle of the light reaching the surface. Hence, under uniform illumination, the shading provides information of the local surface slant relative to the direction of illumination and may thus be used to constrain the interpretation of the relative depth of each point on the surface [1], [2]. Ramachandran [3], [4] utilizing an observation by Brewster [5], [6] discussed by Gregory [7], further showed that the human visual system uses two assumptions in extraction of shape from shading information. One is that there is a single light source illuminating the whole scene, and the other is that the light is shining from above (more precisely, with a slight top-left lighting direction; [8], [9]). With these two constraints, the visual system is able to resolve the shape–from-shading problem, in which a top-to-bottom luminance gradient from bright to dark suggests a convex surface while a gradient from dark to bright suggests a concave surface. Such an effect is often demonstrated with the ‘crater illusion’, in which inverting an image can alter the perception of surface from convex to concave and *vice versa* [5], [10].

The Brewsterian shape-from-shading principle has its limits. As shown in the ‘hollow face illusion’ [11], a hollow face is perceived as a convex regardless of whether the lighting is from above or below. One interpretation for this hollow face illusion is that faces are familiar objects with known shape and thus give little weight to shading information in resolving their 3D shape. Hence, face perception becomes relatively insensitive to shading. This interpretation, however, is in contradiction with a number of studies showing that it is more difficult to recognize or to match bottom-lit faces than top-lit faces [12]–[15]. That is, a change of illumination direction on a face has little effect on the perceived shape from shading but does have an effect on face recognition. In addition it has been shown that face recognition is impaired when the luminance distribution of a face image is contrast-inverted, as in a photographic negative [13], [16]–[18]. However, to the best of our knowledge, there is no reliable measurement on whether observers perceive the same depth from a hollow face or a bottom-lit face as they do on a normal top-lit face. Perhaps only part of the information on those unnaturally lit faces can be recovered by the visual system and the recovered information is enough to perceive a hollow face as convex but not enough for an observer to recognize the shape of a bottom-lit face as well as a top-lit one.

We thus investigated the effect of shading on face perception under a range of illumination directions. To avoid the nuisance of an “unnaturalness” factor that may result from the Brewsterian lighting-from-above constraint, we varied the position of the light source laterally from the front to the side of the face to take advantage of the fact that all horizontal light source positions are ecologically equally plausible.

Since the light that reaches our eyes is a product of both the illumination and the reflectance distribution in 3D space, separating the illumination and face information requires a complex computation in general [19]–[21]. However, for the important case of front-view face images, one can take advantage of the fact that faces are symmetric [22]–[25] to reduce the complexity of the computation [21]. Therefore, in this study we focused on front-view face images. The illumination and face information were separated by an algorithm based on the assumptions that the face is symmetric [22]–[25] with a roughly Lambertian reflectance property and is illuminated by a single light source in addition to the diffuse ambient light (see Methods). Under these conditions, facial information may be partitioned into two types, the information from the inherent coloration and reflectance properties of the facial surface, known as the ‘surface albedo’, and information from the surface illuminance about the 3D face shape, which is the product of the incident illumination and the cosine of the angle of incidence. That is, the net luminance S of a point (x, y) in an image, S(x, y), can be written as the product of these two components:
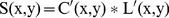
(1)where C’(x,y) denotes the surface reflectance, or albedo, at the location (x,y) while L’(x,y) is the surface illuminance [1],[2],[21]. The prime denotes the absolute levels for these variables, as distinct from the corresponding contrasts introduced without primes below. (For simplicity, we will drop the domain variable (x,y) in the remaining treatment.)

The surface illuminance component L’ is the inner product on the norm vector of the face surface and the direction of illumination [1]. If the surface reflectance C’ is uniform, then L’ is called the reflectance map and Eq. 1 reduces to the bidirectional reflectance distribution function that is commonly used in the classic shape-from-shading problem as discussed by Horn[1],[2]. Thus, in face images, L’ contains the depth information of the face surface.

Notice that neither the reflectance nor the illumination components can have a value less than zero, so we can recast these two components in terms of their contrast variation around the mean (scaled to a maximum of 1).
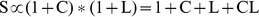
(2)where C is the contrast of the inherent coloration of the face, or reflectance, across the image domain (x,y) and L is the luminance contrast in the image from the illumination component. Notice that C = (C’ − C_m_)/C_m_ and L = (L’ − L_m_)/L_m_, where C_m_ and L_m_ are the mean reflectance and illumination components, respectively.

To the extent that human skin approximates the Lambertian property that its reflectance is proportional to the angle of illumination (except at glossy highlights), the reflectance of any point on the surface at any particular viewing angle is much less than that of the full specular reflectance at that angle. Hence, L in eq. 2 will be close to the maximum only when the illumination direction matches the norm vector of the surface. Thus, given the typical shape of the head, L will be close to the maximum only for isolated spots in the frontal illumination direction. In addition, since most of the face is covered with skin with similar coloration, the surface reflectance C’ should behave similarly and be much less than one in most parts of the face. Some may argue that such simplification may fail in certain parts of a face, such as the eyebrows and eye details that have a different coloration from the skin. However, we computed the size of these details for 76 faces from a database [26] and found that these features combined occupied only about 3% area of the face. Hence, for a face model with no highlights, the product of C and L in the interaction term in Eq 2 can be treated as negligible for our stimuli. Thus, to a first-order approximation,
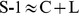
(3)


In general and function may be decomposed into its of its symmetric (S) and asymmetric (A) components. Applying this decomposition to the terms of Eq 3, each term may be decomposed as follows:
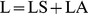
(4a)

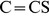
(4b)where LS(x,y) = LS(−x,y) and LA(x,y) = −LA(−x,y) for the illumination component and CS(x,y) = CS(−x,y) for the reflectance component. Notice that the component CA is omitted in Eq. (4b) because the asymmetric reflectance component is negligible (i.e., CA ∼0) in the symmetric views used in the present study. Thus, for a face that is symmetrically viewed, all that is needed for effective segregation of the surface reflectance from the illumination component is to distinguish the residual symmetric illumination component LS from the entire reflectance component CS. We hypothesize that a) CS contains predominantly high-frequency energy whereas LS contains predominantly low-frequency energy and b) the remaining frequency bands in each case (i.e., low frequency CS and high frequency LS) are of little relevance to face reconstruction. This rationale is based on the observation that faces consist predominantly of features with sharp outlines against a background of skin that has predominantly uniform reflectance and smooth changes in shape (at least in younger faces). Hence, we can effectively separate the two symmetric components by a spatial-frequency-specific filter. The important point is that this approach provides a means to vary the perceived depth through the asymmetric shading component of faces without noticeably affecting the featural (albedo) component.

**Figure 1:**
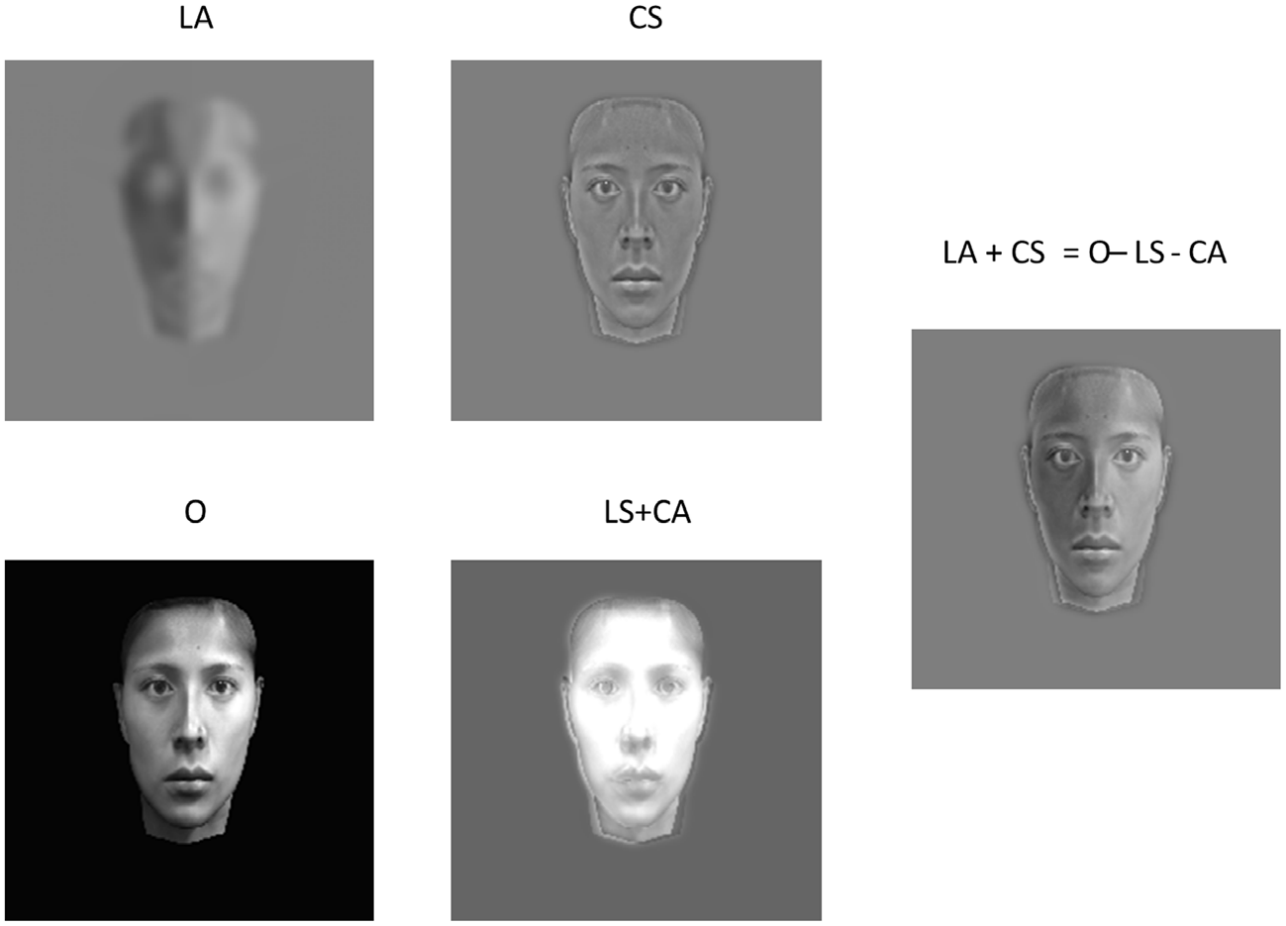
A demo of the dissociation the surface reflectance (CS) from the symmetric and asymmetric illumination components (LS, LA) in an image. Through the process of shape-from-shaping, the LA provided 3D information about a face on a 2D image. The face containing only CS looks very flat. By putting the LA and the CS together, it is easy to see that the hybrid image (LA+CS) and is similar to the original (O) even though a lot of information (LS+CA) was thrown away. The original images (O) were created from Lin et al. (2002, 2005).

Figure 1 demonstrates our method. LA is the asymmetric low spatial frequency component that represents the asymmetric illumination information of the original face image, O, while CS is the symmetric high spatial frequency that represents the reflectance component. The hybrid face LA+CS is the sum of the LA and CS and is perceptually almost equivalent to the original face. This demonstration illustrates that the combination of the residual *asymmetric* high frequency and the *symmetric* low spatial frequency components (LS+CA) plays little role in face perception. Hence, subtracting them from the original face image hardly affects our percept.

**Figure 2:**
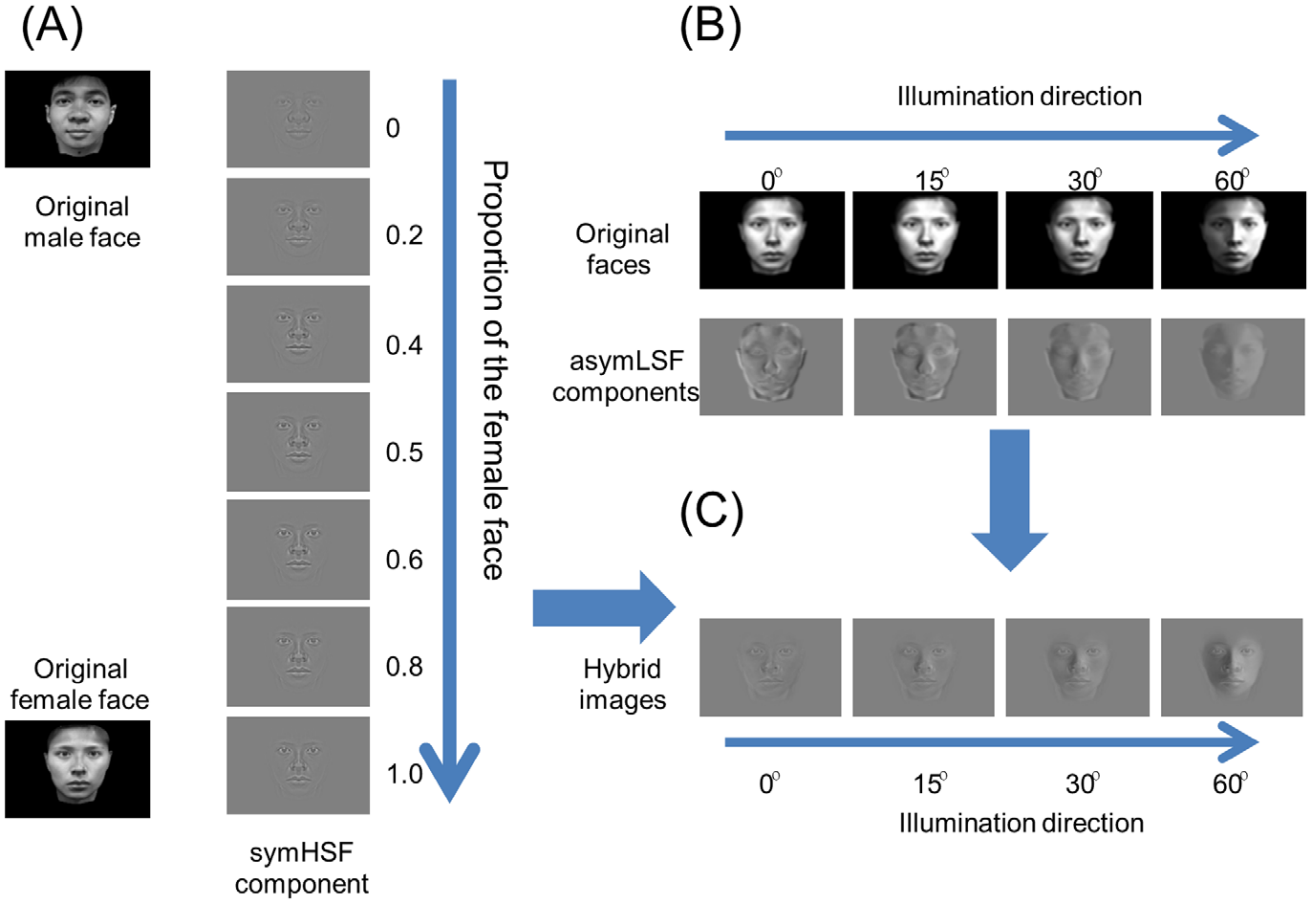
Examples of the low and high spatial frequency hybridization. A, A series of intermediate face examples were generated by morphing the symmetric components of high special frequency (symHSF) between the two faces, while holding the low spatial frequency information invariant. There were seven levels of this component with proportion of the female face from 0 (entirely male face) to 1 (entirely female face). B, Examples the low spatial frequency (asymLSF) components from the female face after low-pass filtering the original face to preserve coarse-scale shading information, computed for four illumination directions: 0°, 15°, 30°, and 60°. C, The hybrid face stimuli were a combination of the asymLSF female face (or male face, not shown) of one illumination direction on the morphed face. Here examples are shown with the 0.5 female symHSF component, combined with a female asymLSF component at the four lighting directions, which can be seen to enhance the perceived depth as the illumination angle increases. The resulting hybrid face at the 60° illumination angle was categorized as female by most observers. The images were rendered from 3D models by Lin et al. (2002, 2005). The models have given written consent to publication of their photos.

In the current study, we investigated how the change of illumination in terms of the asymmetric low spatial frequency component affects a) face discrimination and b) depth judgments on hybrid faces. The hybrid picture paradigm [27], [28] was employed to study the relative contributions of specific spatial frequency ranges. In our case, the hybrid face is a combination of CS and LA or CS and LS (Eq 1–3). Specifically, we morphed the symmetric high spatial frequency component of a male face with that of a female one with different weightings to generate a set of high spatial frequency components that contained information from both faces (Figure 2a). We then added the low spatial frequency components from a range of illumination directions (Figure 2b) to create hybrid faces (Figure 2c) of a range of gender mixes and illumination directions. We also included examples of symmetric low spatial frequency content as controls.

The goal of the experiment was to measure the face discrimination performance for these hybrid faces. If the illumination direction affects the retrieval of 3D shape information from a face, we should expect the differences among illumination conditions to affect both face discrimination and depth judgment for the hybrid faces. In addition, controls were included to test the proposal that such an illumination effect should exert its influence through the asymmetric low spatial frequency component of the face images with little contribution from the symmetric component.

## Methods

### Ethics Statement

This study was approved by the Institutional Review Board of National Taiwan University Hospital. Written consent was obtained from each observer. The use of human participants follows the principles expressed in the Declaration of Helsinki and local regulations.

### Apparatus

The stimuli were presented on a ViewSonic VA902 17″ LCD monitor controlled by a HP D325MT computer with an ATI Radeon 9800PRO graphics card running at a temporal refresh rate of 60 Hz (non-interlaced) with a spatial resolution of 1280(H)×1024(V). The viewing distance was 127.5 cm, at which each pixel subtended about 0.01° (H)×0.01° (V). In order to provide a fully linearized luminance output, the gamma function of the monitor was calibrated to 1% precision with a LightMouse photometer [29], and this information was used to compute a linear 8-bit color look–up table. The accuracy of the resultant look-up tables was verified by an international Light RPS-380 spectroradiometer. The experimental control software was written in MATLAB (MathWorks Inc, 1993) in conjunction with the Psychophysics Toolbox [30],[31]. The display had a mean luminance of 15 cd/m^2^ and a chromaticity of (0.33, 0.33) in CIE 1931 xy coordinates.

### Stimuli

The face images (Figure 2) were constructed from a pair of 3D laser-scanned faces developed by Lin and colleagues [32],[33], consisting of a 3D face model of one female face and one male face with neutral expressions. (Only one face of each gender was available in the 3D model format required for our shading manipulations.) The front-view face images were rendered from the textured 3D models using 3ds Max 2009 (Autodesk, Inc., 2009). The Blinn–Phong shading model [34] was employed to construct the requisite illumination direction. We first placed four diffuse light sources around the model to produce an even ambient light and keep the shadowed regions from being too dark. A point light source was specified at 0°, 15°, 30°, and 60° relative to the center of the head and on the horizontal meridian of the faces (where an illumination direction of 0° means that the light source is directly in front of the face). The point light source and the ambient light were set at an intensity ratio of 7.5.

In addition, the 3D face models under ambient illumination were used for constructing purely symmetric high spatial frequency stimuli for the hybrid face generation as described below. All images were grayscale images with 256 gray levels.

As developed in Eq. 3a and 3b, symmetrically-viewed face images can be decomposed in theory into three parts: asymmetric and symmetric illumination components LA and LS, and a symmetric reflectance component CS. This decomposition was achieved in practice by the following steps. First, the face images were Fourier transformed to the frequency domain and the real and imaginary parts of the transformed images taken as defining the symmetric and asymmetric components, respectively. Both the symmetric and asymmetric components were multiplied by a) a low pass (N (0. σ)) and b) a high pass (1-N (0, σ)) Gaussian-defined function, where the cut-off frequency σ was set to 12 cycles per face width, to extract the low and high spatial frequency information, respectively. An inverse Fourier transform then allowed the face images to be separated into four components: Symmetric low spatial frequency (symLSF, corresponding to LS in Eq. 3a), symmetric high spatial frequency (symHSF, corresponding to CS in Eq. 3b), asymmetric low spatial frequency (asymLSF, corresponding to LA in Eq. 3a), and the asymmetric high spatial frequency (asymHSF) component. All the components of all images were normalized to have equal RMS contrast before further processing.

As demonstrated in Figure 1, the asymHSF information has little influence on 3D face perception. Therefore, this component was not used in the main experiments. The symHSF images, which look relatively flat, convey detailed edge information of facial features and in turn the precise 2D shape of the eyes, nose, mouth, eyelashes, etc. By contrast, the LSF face images provide a strong ‘sculptural’ depth impression and represent the coarse-scale 3D facial configuration and illumination information.

The hybrid face stimuli we used were a combination of a) a symHSF only image, constructed from face images created under ambient illumination conditions, and b) either a symLSF or an asymLSF component of the face image created under point lighting conditions. We used FantaMorph 4.0 (Abrosoft, 2009) to generate image morphs between the male and female symHSF faces. There were seven morphed symHSF images with the proportion of the female face varying between 0 (entirely the male face) and 1 (entirely the female face). Figure 2A shows examples of these symHSF image morphs.

The asymLSF components were created from faces rendered at four horizontal angles of illumination (0°, 15°, 30° and 60°). The rendered faces and their asymLSF components under the four illumination angles are illustrated in Figure 2B. To ensure that the symLSF component, which is invariant to lateral changes asymmetric illumination, has little effect on face discrimination, we also included this symLSF component of the face under the 60° illumination as a control.

Finally, the hybrid faces (Figure 2C) consisted of the neutral symHSF component at a particular male-to-female morphing level and either the asymLSF from one illumination condition or the (control) symLSF component at the high illumination angle. The LSF components were from either the 100% male or the 100% female faces. In total, there were 56 (4 asymLSF illumination directions x 2 faces (male and female) × 7 symHSF morphing levels) hybrid face images used in the experiment. In addition, for further controls, we also used 14 symLSF and symHSF hybrid images (2 faces (male and female) x 7 symHSF morphing levels) and 7 symHSF-only images at different morphing levels. Hence, a total of 77 face images were used for the study. Finally, the constructed hybrid images were windowed with a 2D 4-th power Gaussian function exp(-x^4^/s_x_^ 4^-y^4^/s_y_^4^), where s_x_ = 1.2° and s_y_ = 1.3° were the space constants for the x and y dimensional respectively. The 4-th power Gaussian has a nice feature of retaining almost all information within 1 unit of the space constant but removing much information beyond this range with a narrow but smooth transition. Hence, given that the ear-to-ear width of the face was set at 3.0°, this filtering retained all the face features but removed irrelevant external cues such the hair, ears, and outer contours of the faces.

### Procedure

#### Face discrimination

We used the method of constant stimuli to measure the proportion of ‘female’ judgments for each test image. On each trial, the observers were presented with one of the 77 face images for a duration of 500 ms. The order of presentation of the images was randomized. After the presentation, the observers were shown a blank screen at the mean luminance and asked to press a key to indicate whether the stimulus was perceived as more similar to the male or female face. The next trial began 1 s after the observer responded. Observers were not given feedback about their performance.

Each psychometric function consisted of the proportion of “female face” judgments on images of the same illumination condition but at different male-female morphing levels. There were 40 trials per face image. Hence, with seven morphing levels, there were 280 trials per psychometric function. In the main experiment, given 8 asymLSF+ symHSF (4 illuminations at 0°, 15°, 30°, and 60°, x 2 individual faces), and one symHSF-only condition, this design generated a total of 11 psychometric functions for each observer.

Furthermore, to assess the effect of the symLSF component, one control experiment provided two extra psychometric functions (male and female asymLSF from the 60° illumination condition) for a comparison with the corresponding asymLSF+symHSF psychometric function from the main experiment. To assess the effect of the asymHSF component, a further experiment provided the psychometric function with the asymHSF-only stimuli to be compared with the symHSF only condition of the main experiment. All the test conditions are listed in Table 1.

**Table 1:** Stimuli used in the experiments.

	Conditions	Description
Main experiment		
	SymHSF Only	The symmetric high spatial frequency component. The base line condition.
	AsymLSF+SymHSF	The sum of symHSF and the asymmetric low spatial frequency components with 0°, 15°, 30°, or 60° illumination.
Control Exp 1: The effect of the symmetric low spatial frequency component		
	SymLSF+SymHSF	All the symmetric components from the images with 60° illumination.
	AsymLSF+SymHSF	The sum of symHSF and the asymmetric low spatial frequency components with 60° illumination from the main experiment.
	SymHSF Only	The baseline condition from the main experiment.
Control Exp 2: The effect of the asymmetric high frequency component		
	AsymHSF only	The asymmetric high spatial frequency component
	SymHSF only	The baseline condition from the mail experiment.

#### Depth judgments

In the second experiment, the observers were asked to judge the perceived depth of the face images relative to the depth of a fixed reference image of a shaded sphere. The face images used for the depth judgments were at the 0 or 1.0 morphing levels of those used in the face discrimination. The reference spheres were created with the 3ds Max 2009 software package (Autodesk, Inc., 2009) with the diameter of the sphere matching the width of the face images and a symmetric (0°) illumination direction.

In each trial, observers were presented with one of the face images together with the reference sphere for 1 s. The face and the sphere were presented adjacent to each other, separated by a 3′ gap. The location (left or right of the fixation) of the face and the sphere was randomly determined. The observer had to press one of the keys to rate the relative depth of the faces using a 5-point Likert scale (1– flat, 5– very deep), anchored by the specification that the reference sphere should be considered to have a depth of 3 on this scale.

### Participants

Seven observers, all with normal or corrected-to-normal vision, participated in this study and were financially compensated for their time. All were naïve to the purpose of this study and were not familiar with the people whose faces were used as stimuli. All observers were Taiwanese recruited from National Taiwan University campus. Hence, there should be no other-race effect [35], [36] involved. Informed consent was obtained from each observer before the experiment.

### Data Analysis

For each LSF condition, the fraction of female face responses was plotted against the morphing level of the symHSF component. The psychometric functions were fitted with a cumulative Gaussian function a+(1 − *a* − *b*)* Φ(*x; μ, σ*), where *μ* is the location parameter (mean) of the Gaussian, and hence specifies the point of subjective equality (PSE), *σ* determines the slope of the psychometric function, and *a* and *b* define the lower and upper bounds of the psychometric function, respectively. The significance of the differences between PSEs for different conditions was evaluated with a two-tailed paired *t* test, taking Bonferroni corrected p<0.05 as the criterion for significance.

## Results

### How asymLSF Information Changed the Perceived Facial Information of Hybrid Faces

**Figure 3:**
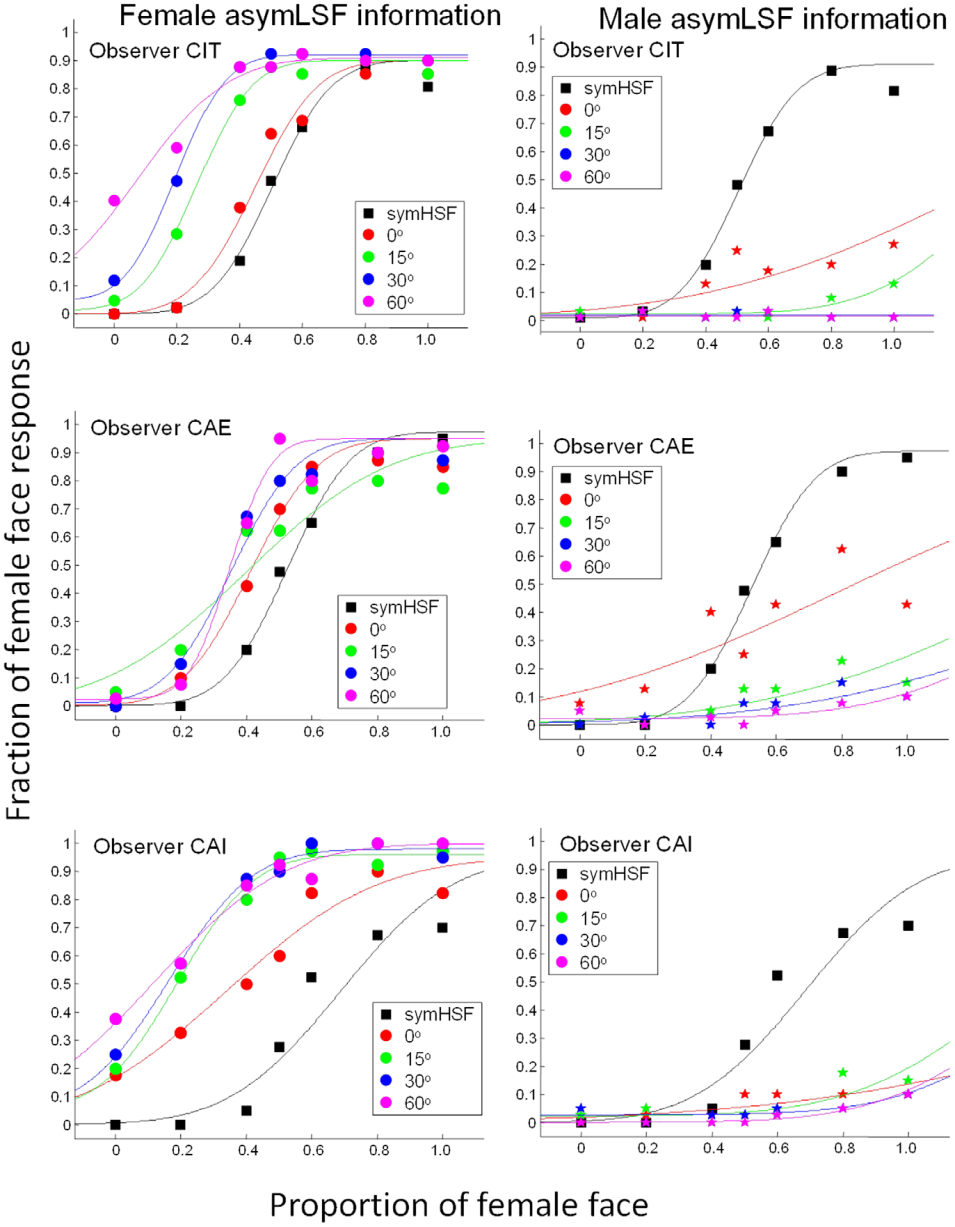
The effect of asymLSF on face discrimination. Each row shows the psychometric functions from one of the three naïve observers. The fraction of “female face” responses is plotted as a function of the proportion of female face in the combined images. The black psychometric function is for the symHSF-only condition plotted here for comparison. The red, green, blue and magenta psychometric functions represent different illumination directions as indicted in the legend. In the female-face asymLSF condition (left column), the psychometric functions shifted gradually to the left as illumination angle increased. Conversely, in the male-face asymLSF condition (right column), the psychometric functions shifted dramatically to the right.

Figure 3 shows the fraction of female face judgments from three representative observers as a function of morphing level for the symmetric high spatial frequency (symHSF) component, for hybrid faces with asymmetric low spatial frequency (asymLSF), separately for the 0°, 15°, 30°, and 60° illumination directions. The left column shows the psychometric functions measured with the asymLSF components from the female face, while the right, those from the male face. The psychometric functions for the symHSF-only condition (black squares and curves) are plotted in the panels for the corresponding observers as a comparison.

In the symHSF only condition, the psychometric functions show a sigmoid shape. The proportion of female response increased monotonically with the proportion of female components in the image. The neutral symHSF yielded about 50% “female” response. Hence, there is little, if any, bias in response toward any particular gender in the symHSF-only condition.

Adding the asymLSF component from the female face to the symHSF component shifted the psychometric curves to the left. The amount of change generally increased with illumination direction. The observers saw the hybrid faces as tending towards the female face even when the symHSF contained a very small proportion of that face information. Conversely, adding the asymLSF component from a male face shifted the psychometric functions dramatically to the right. This rightward shift was especially pronounced with the 30° and 60° illumination directions, in which the observers always saw the male face regardless of the morphing level. Thus, the asymmetric low spatial frequency information from the male face had a stronger effect than that from the female face. This bias may be due to the difference in gender information between the two faces, since Bruce et al. [37],[38] showed a similar male advantage effect in face recognition and discrimination tasks.

**Figure 4:**
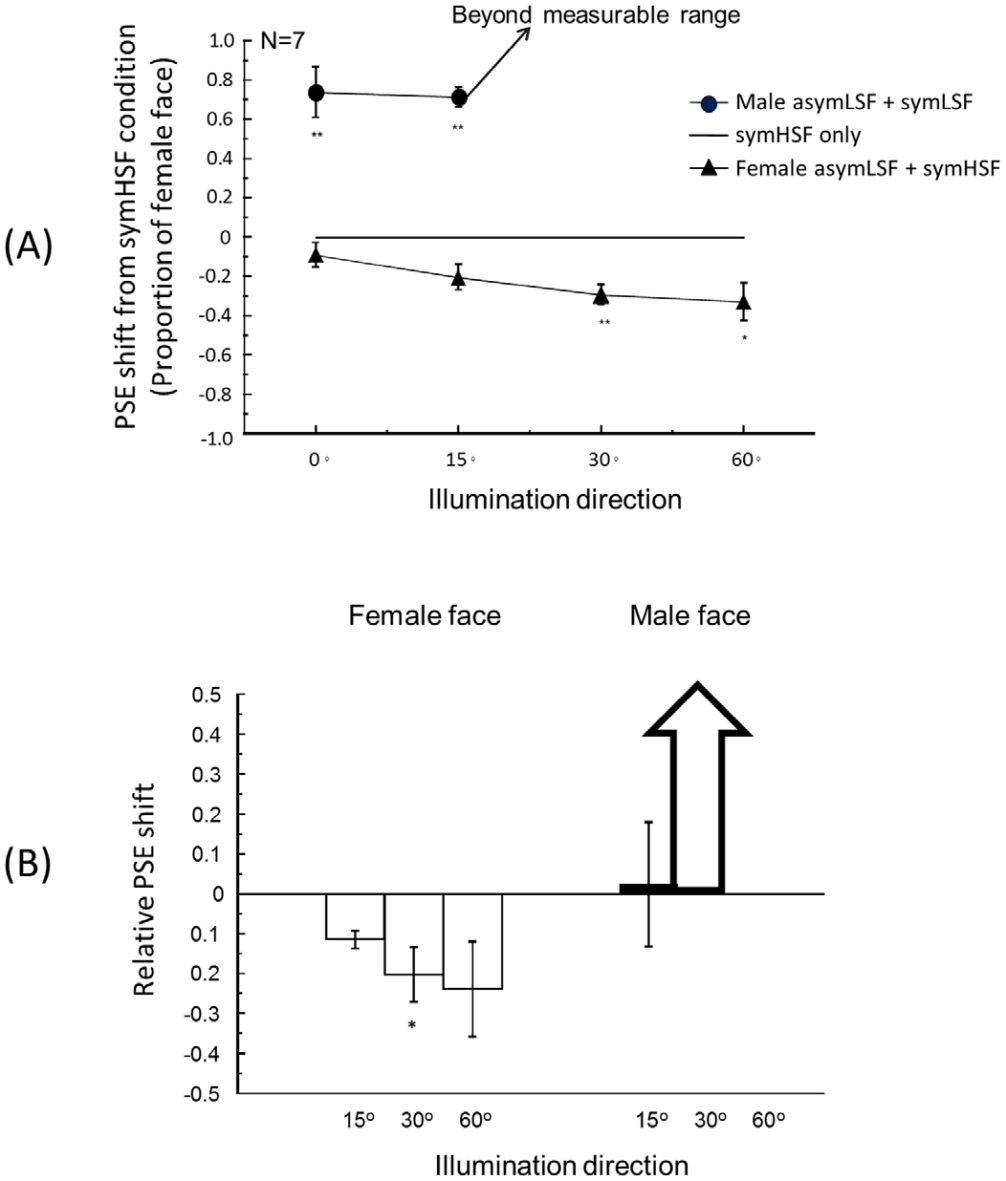
PSE shift. (A) AsymLSF effect on PSE shift from the symHSF only condition averaged across observers. (B) Relative PSE shift from the 0° lighting direction. The error bars represent one standard error. The statistically significant difference at p<.05, and 0.01 in a two-tailed paired t-test after Bonferroni correction are indicated by one, and two asterisks respectively. The averaged PSE shifts at male face 30° and 60° illumination directions were so great that they were beyond the measurable range, as indicated by the arrows.

To quantify the effect of asymLSF and summarize the results from all seven observers, we determined the PSE (the proportion of femininity corresponding to 50% female responses) for each psychometric function of each observer. Figure 4(A) shows the mean PSE relative to the symHSF-only condition. A positive value means a rightward shift of the psychometric curve, or a bias toward more male-face responses relative to the symHSF-only condition, while a negative value means a leftward shift or a bias toward more female-face responses. The *p-*value shown in Figure 4 was a two-tailed paired *t* test comparing the PSE of each illumination condition with the symHSF only condition. The error bars show ±1 standard error of the means (SEM) across observers. All asymLSF effects were significant except for the female-face asymLSF at the 0° illumination direction. In addition, the PSE shift increased with lighting direction (Figure 4B). For the male face, even though the residual shading information from the complicated 3D structure face allowed a significant asymLSF effect, the asymLSF shifts from the 30° and 60° conditions showed significant enhancements of the discrimination that were so effective that their PSEs were beyond the measurable range. Hence, the more extreme illumination angles made it easier for the observers to bias their response toward the gender of the asymLSF component.

### Perceived Facial Information of Hybrid Images is Invariant with Symmetric LSF Information

**Figure 5:**
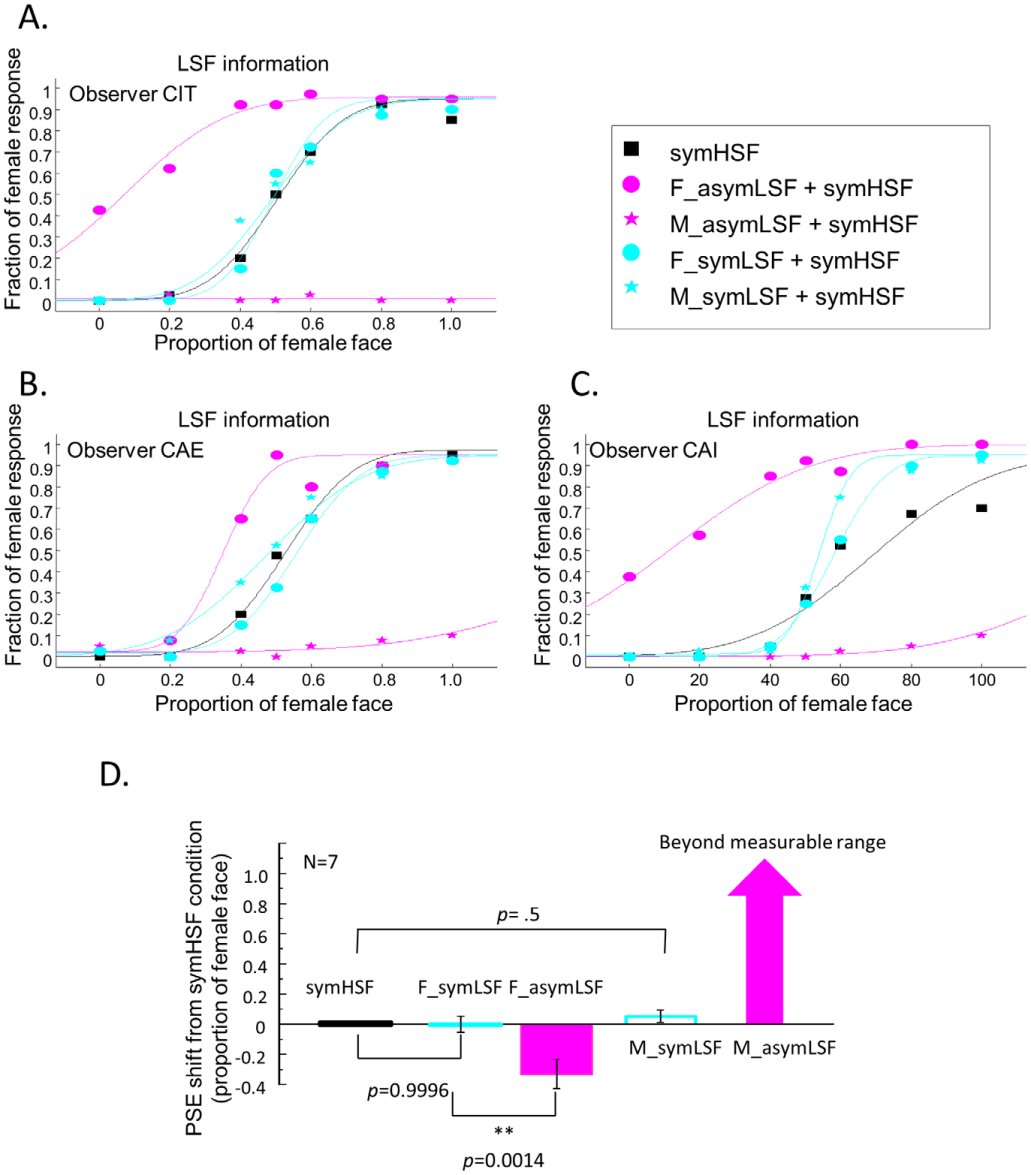
Comparisons of the effect of the asymLSF and symLSF information on face discrimination. A-C, Psychometric functions from three naïve observers. The fraction of “female face” responses is plotted as a function of the proportion of female face. Black curves: symHSF-only condition in the corresponding observers. Cyan curves: symLSF+symHSF in the female and male conditions. Magenta curves: asymLSF+symHSF faces. The symLSF component had very little influence on face discrimination, as indicated by the fact that the psychometric curves are not significantly shifted. D, The average PSE shifts relative to the symHSF-only condition. Other conventions as in Figure 3.

We also investigated whether perception of the face stimuli could be influenced by the low-frequency ***symmetric*** component (symLSF) in the way that the asymLSF component does. Panels A–C of Figure 5 shows the results for three representative observers in judging the face hybrids consisting of the fully symmetric combination of symLSF and symHSF information (cyan symbols and curves). The psychometric functions for the asymLSF conditions of the same 60° illumination condition (magenta symbols and curves) and the symHSF only condition (black symbols and curves) are replotted here for comparison. Star symbols denote the judgments for the male-face and circles the female-face LSF components, respectively.

In contrast with Fig. 3, the symLSF information produced no significant effect on the psychometric functions even though it was derived from either a 100% male or 100% female face. Figure 5D summarizes the PSEs from the seven observers. Relative to the symHSF only condition, the addition of symLSF information produced no significant bias (*t*(6) = −0.00045, *p*>.99 for the female-face condition and *t*(6) = −1, *p = *.5 for the male-face condition). On the other hand, the difference between the asymLSF and symLSF conditions was highly significant (*t*(6) = 5.603, *p = *.0014 for the female-face condition, and the difference between them for the male-face condition was obviously significant but beyond the measurable range. That is, adding asymLSF components biases the observer’s responses while adding symLSF components did not have such effect.

Some may argue that testing the effect of the symLSF at 60° illumination may not be ideal. After all, the intensity of symLSF might be too weak for a fair comparison. However, since we normalized the contrast of each component (see Method), the intensity of the symLSF and asymLSF components should be equated. Furthermore, even without normalization, the symLSF at 60° illumination should retain half of the intensity of that at 0° illumination. Hence, the shape information that might be in symLSF should remain intact and thus would not distort the evaluation.

**Figure 6:**
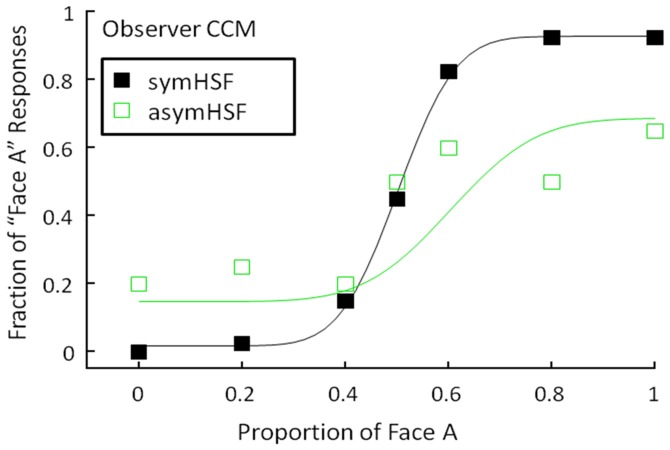
A Comparison of the effect of asymHSF and symHSF on face discrimination. The fraction of “female face” responses is plotted as a function of the proportion of the female face in the shading image. Black curve and closed squares: the symmetric HSF condition. The green curve and open squares: the asymmetric HSF condition. Other conventions as in Figure 3.

As a further control, we made the same comparison for the HSF components. Figure 6 shows the asymHSF and symHSF psychometric functions for a representative observer. The symmetric HSF psychometric function (black closed squares) showed the same behavior as those in Figures 3 and 5: the proportion of female face responses increased with the proportion of the female face in the shading image, from essentially all “male face” responses at a 0.3 female-face ratio to essentially all “female face” responses at a 0.7 ratio.

In the high spatial frequency range, on the other hand, the asymmetric HSF condition (green open squares), produced a psychometric function with much shallower slope. There was a substantial proportion of “female face” responses even for images with no female face content, and *vice versa*. This weak variation with the female face level suggests that the asymHSF component biased perceived face less than the symHSF component.

### Depth Judgments

**Figure 7:**
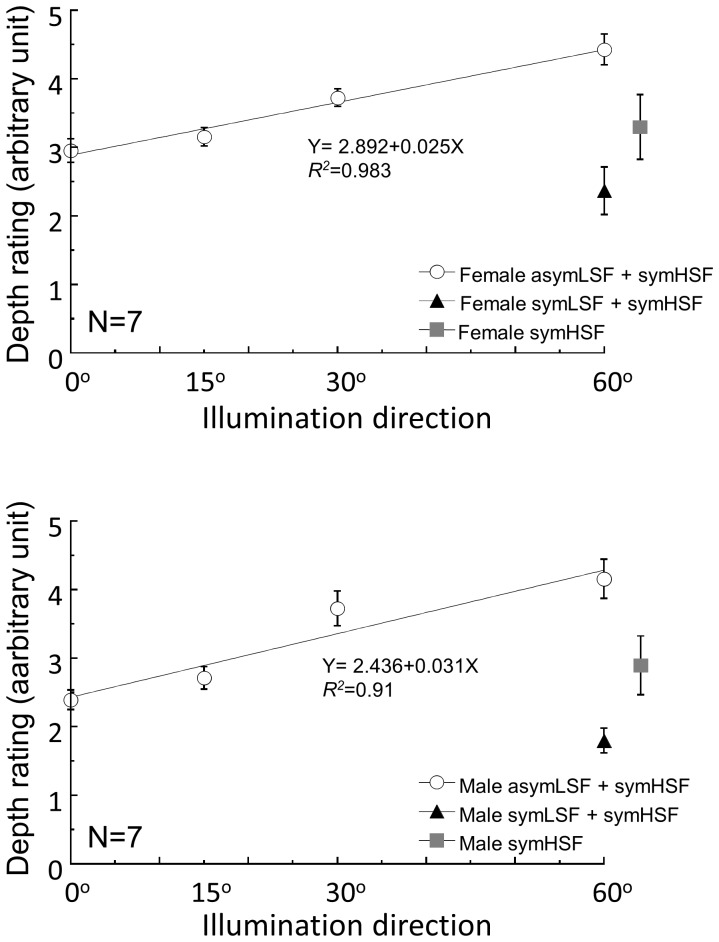
Depth judgments Depth judgments. The depth rating value of the hybrid faces: asymLSF (under four illumination directions) combined with symHSF, symLSF combined with symHSF (under 60°), and female (A) and male (B) symHSF. The depth value of the reference sphere was set to 3. Using least squares analysis, the linear regression lines of asymLSF condition of both faces show a positive increase in proportion to the illumination direction.

Figure 7 shows the depth ratings for the asymLSF+symHSF (open circles) faces from the four illumination directions. The depth ratings for the symLSF+symHSF (closed triangles) faces and the symHSF-only (closed squares) faces are plotted for comparison. The symHSF components were set at either 1.0 (female face, Panel A) or 0.0 (male face, Panel B). The depth value of the reference sphere was set to 3. The perceived depth for the asymLSF+symHSF faces increased from the baseline level with the horizontal direction of lighting. This increase is well described by a linear regression line. The linear regressions were highly significant (*p*<0.001) with *R^2^* at 0.983 and 0.91 for the female and male asymLSF+symHSF hybrid faces, respectively. The slope parameters were significantly larger than zero (*t*(2) = 10.67, p = 0.0086 for the female face and t(2) = 4.49, p = 0.046 for the male face).

This result shows that the change in depth judgments was consistent with the PSE shift with different illumination directions. That is, as the illumination direction shifted to the side, the asymLSF had a greater effect not only on face perception, but also on the perceived depth of the faces. Conversely, the perceived depth of the symHSF-only face was about the same as the 0° asymLSF+symHSF (*t*(6) = 0.86, *p = *0.42 for the female-face condition and *t*(6) = 0.91, *p = *0.39 for the male-face condition) while that for the symLSF+symHSF was about the same as or lower than that for the asymLSF+symHSF faces. Thus, the symHSF information carried no significant depth information, as we originally hypothesized from eq. 3.

### Generality of the Results

Finally, we note that our interpretation is based on the performance of the observers to the images in our particular stimulus set. The judgments might have been made by the perceived gender, the structural identity, or both, of the face stimuli. Hence, the effect of limiting the observer responses solely to perceived gender or perceived identity as single variables across an extended stimulus set cannot be inferred from the results of our main experiment.

**Figure 8:**
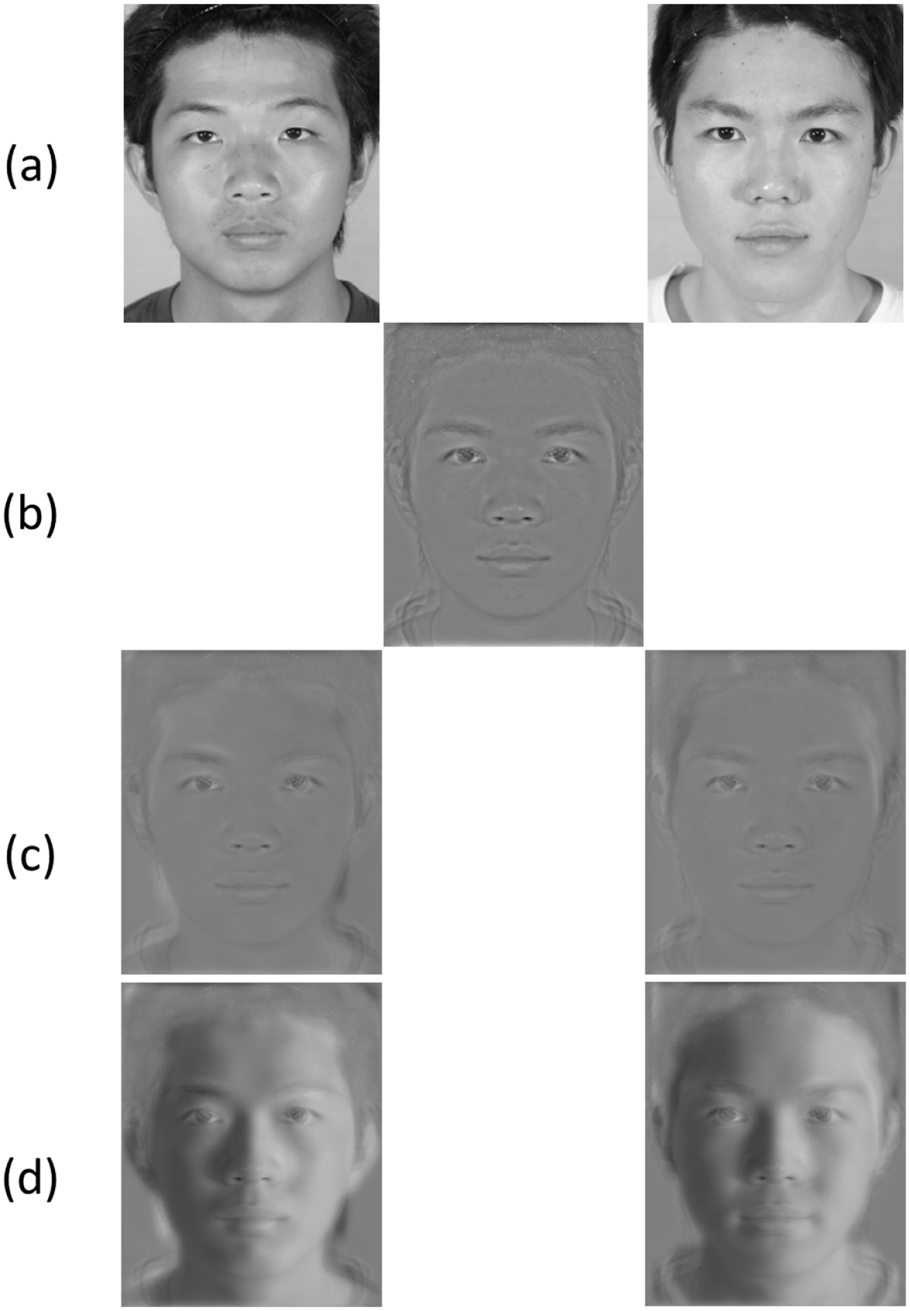
The shading effect holds for faces of the same gender. (a) The original images of two male faces were from a publicly available database prepared by Shyi et al. (2011). (b) The 50–50 morph of the symHSF components of the two faces. (c) The combination of (b) and asymLSF components of 0° lighting images. These two reconstructed images look very similar. (d) The combination of (b) and asymLSF components of 45° lighting images. These two images are clearly from two different individuals. The models have given written consent to publication of their photos.

One way to resolve this problem would be repeat our experiment with face images from many individuals. However, since, at the time of writing, the technology for 3D modeling of real human face remains immature and expensive, it is impractical for us to get more 3D head models. We, however, were able to get further face images with zero and 45 degree illumination from a recently available image database [39]. We then selected two pairs of male images (Figure 8a) from this database and then processed the images with the same method as that in our main experiment. We extracted the symHSF component from the 50–50 morphed image (Figure 8b) and combined it with the asymLSF component from either 0° or 45° illumination of the two individuals. The stimuli are shown in Figure 8(b) and (c). As shown in Figure 8, the two images reconstructed with the asymLSF components from 0° lighting of the two individuals look rather similar. However, those with 45° aymLSF components are clearly images of two different persons. We tested them on seven observers. The task of the observers was to press a key to indicate which person they perceived. Other details of the experimental procedure were the same as those in the main experiment.

**Figure 9:**
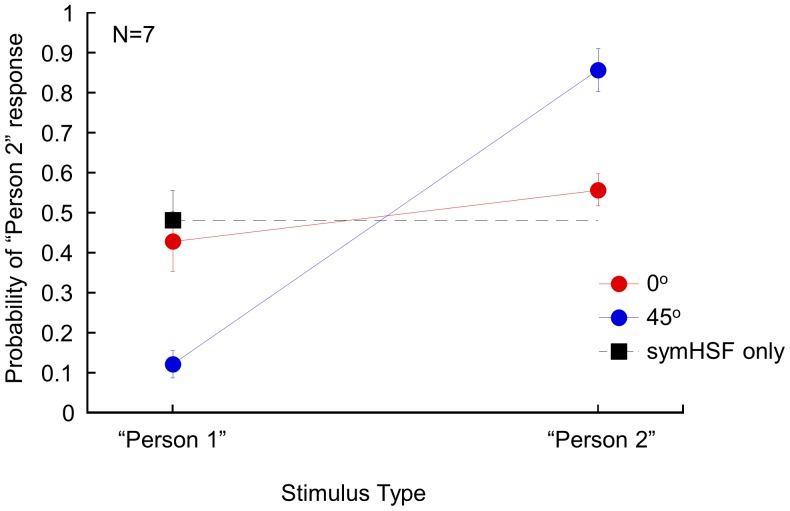
The effect of asymLSF on discriminating two male faces. The fraction of “Person 2” responses is plotted against the source of asymLSF component. The black square and dash line is for the symHSF-only condition plotted here for comparison. The red and blue circles represent 0° and 45° illumination directions as indicted in the legend.

Figure 9 shows the results. The observers were ambivalent about the identity of the symHSF only stimulus (48% “Person 2” response, black square and dashed line). This is not surprising as it was from a 50–50 morphed face. The asymLSF from 0° illumination (red circles) provided little help as the “Person 2” response was 0.43 (t(6) = −0.94, p = 0.38) and 0.56 (t(6) = 0.20, p = 0.20) and was not significantly different from the chance level with Person1 and Person 2 asymLSF component added. On the other hand, the observers had little problem in telling the difference between the two faces with asymLSF component from 45° illumination (blue circles). The probability of “Person 2” response was 12% with Person 1 asymLSF (t(6) = −11.05, p<0.001) and 86% with Person 2 asymLSF (t(6) = 6.62, p<0.001). Hence, even with non-optimal stimuli, we still get the same effect as in the main experiment. Thus, this shading effect on face discrimination is quite robust. In addition, as in Figure 8, this shading effect also holds for face images from two males. That is, the result implies that it is an effect of face identity discrimination, not just gender discrimination.

## Discussion

Our results conformed to the analytic hypothesis of Eq 1–3 in showing that asymmetric lighting information (asymLSF) had a profound effect on face perception, as it greatly biased the percept of the hybrid faces in the observers. That is, the asymLSF component from a female (male) face made the observers more likely to perceive the hybrid face as female (male). In addition, the effect of the asymLSF component increased with illumination direction. However, the symmetric lighting (symLSF) component had no such effect. Moreover, in accord with our original hypothesis, the asymLSF component had a corresponding effect on the perceived depth of hybrid faces that increased in proportion to the shift of the illumination direction to the side, whereas the symHSF component had little effect on perceived depth.

### Illumination Effects

The local interplay between surface orientation and source of illumination generates shading gradients on objects. Consequently, the shading pattern should be affected by a change in the illumination direction. Since shading provides incomplete information regarding 3D shape [2], a different illumination direction could result in changes in the cortical face representation via shading information [40], as our results suggest.

In addition to the aforementioned studies investigating the differential effect of top-lit and bottom-lit faces [12]–[15], [41], it has been shown that varying the azimuthal illumination angle impairs face recognition memory [14], [40]–[42]. That is, it is more difficult to recognize a face when the illumination in the learning phase and in the test is different. In combination, these studies support the idea that the illumination direction plays an important role in face perception. Besides, the effect of light source position on perceived depth also has been investigated for non-face objects. The perceived curvature of a spherical surface increases with increasing slant of the light source [43].

**Figure 10:**
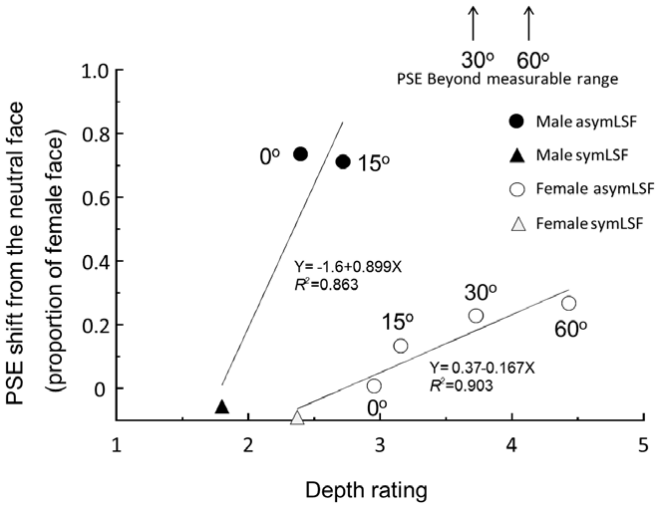
Face discrimination performance is highly correlated with perceived depth. Pearson correlation coefficient of depth rating value and averaged PSE shift was *r = *0.90 for the female-face and *r = *0.86 for the male-face conditions The averaged PSE shift in the male-face 30° and 60° illumination conditions was beyond measurable range, as indicated by the arrows.

Our results are consistent with these previous studies. We have shown that manipulation of illumination direction to change the shading information on faces biases *both* the depth perception of faces *and* the face discrimination performance. Figure 10 shows that the PSE shift in Figure 3 is highly correlated with the bias in perceived depth (Pearson correlation coefficient, *r* = 0.90 for the female face condition, and *r* = 0.86 for the male face condition). While the physical depth of the face model remains the same, increasing the effective shading information by moving the illumination direction to the side helps the observers to better perceive depth in a face presented on a flat screen, as implied by the greater perceived depth ratings. This result supports our hypothesis that increasing the depth percept improves the ability to resolve the depth structure of the faces, and consequently leads to an enhanced ability to discriminate fine cues to the gender of those faces. The data also exhibit the corollary that, if the illumination direction does not provide appropriate shading information to recover sufficient depth information, it is more difficult for an observer to perceive the faces accurately.

### Spatial Frequency Effect

There is considerable evidence indicating the importance of coarse-scale facial information in face recognition. The pioneering work of the ‘Lincoln illusion’ demonstrated by Harmon & Julesz [44] showed that, in pixelized portraits rendered unrecognizable by the high frequency masking introduced by a sampling and quantizing procedure, recognition was improved.applying a low-pass filter to remove the high-frequency components. Moreover, they suggested that low spatial frequency information is more critical for face recognition than high spatial frequency information. Harmon & Julesz suggested that a spatial frequency as low as 2.5 cycles per face width was sufficient for face identification. Several further studies have shown that face recognition is optimal at medium spatial frequencies from 8 to 16 cycles per face width [45]–[51]. These results indicated that removal of low to medium spatial frequencies has a more detrimental effect on face recognition than removal of high spatial frequencies. Our finding that asymmetric information beyond 12 cycles per face plays little role in face perception is generally consistent with these findings of the importance of low and mid spatial frequencies.

While it is generally agreed that the mid spatial frequency information is critical for face recognition, the perceptual mechanism by which this information is processed has not been previously investigated. Thus, unlike the aforementioned reports, we went beyond the investigation of purely spatial frequency range effects in face perception to consider their role in the perception of the 3D structure of the face. The specific novelty of our study is to identify that the shading information, and in turn the 3D structure carried by the asymmetric low spatial frequencies, is critical in face perception for the azimuthal illumination directions used in our experiments. This notion is supported by our result that (1) the asymmetric low spatial frequency information dramatically affects both perceived facial information and perceived depth; (2) this effect increases as the illumination direction is shifted to the side and (3) adding the symmetric low frequency information had little effect on facial discrimination, implying that the asymmetric effect on face perception could not be attributed to the luminance contrast *per se*. Thus, the data are consistent with our hypothesis that the critical role of low spatial frequency information for face perception is due to the depth structure information derived from shape-from-shading.

### Conclusions

This study was designed to test the role of the perceived depth derived from asymmetric low spatial frequency information in face images. Face perception in relation to the perceived depth was determined with a hybrid image paradigm and face images constructed under a range of illumination directions. The strong influence of the asymmetric low spatial frequency information on face perception is illustrated by its ability to change the percept of observers on for hybrid face. In addition, this effect increased with the angle of the illumination direction.

In contrast, the symmetric (front-lit) low spatial frequency information had little, if any, influence on face perception. In evaluating the hypothesis that the mechanism for these effects lay in the change of the perceived depth evoked by the lighting component, we found that the perceived depth of a face became more pronounced with the degree of asymmetric low spatial frequency information as the illumination direction shifted to the side. Taken together, these results are consistent with the notion that the sideward shift of the illumination direction provides more shaded area on faces, which increases the perceived depth of shape-from-shading and in turn changes the facial perception ability of the observers.
